# Learning of Arbitrary Association between Visual and Auditory Novel Stimuli in Adults: The “Bond Effect” of Haptic Exploration

**DOI:** 10.1371/journal.pone.0004844

**Published:** 2009-03-16

**Authors:** Benjamin Fredembach, Anne Hillairet de Boisferon, Edouard Gentaz

**Affiliations:** CNRS and University of Grenoble 2, Grenoble, France; University of Sydney, Australia

## Abstract

**Background:**

It is well-known that human beings are able to associate stimuli (novel or not) perceived in their environment. For example, this ability is used by children in reading acquisition when arbitrary associations between visual and auditory stimuli must be learned. The studies tend to consider it as an “implicit” process triggered by the learning of letter/sound correspondences. The study described in this paper examined whether the addition of the visuo-haptic exploration would help adults to learn more effectively the arbitrary association between visual and auditory novel stimuli.

**Methodology/Principal Findings:**

Adults were asked to learn 15 new arbitrary associations between visual stimuli and their corresponding sounds using two learning methods which differed according to the perceptual modalities involved in the exploration of the visual stimuli. Adults used their visual modality in the “classic” learning method and both their visual and haptic modalities in the “multisensory” learning one. After both learning methods, participants showed a similar above-chance ability to recognize the visual and auditory stimuli and the audio-visual associations. However, the ability to recognize the visual-auditory associations was better after the multisensory method than after the classic one.

**Conclusion/Significance:**

This study revealed that adults learned more efficiently the arbitrary association between visual and auditory novel stimuli when the visual stimuli were explored with both vision and touch. The results are discussed from the perspective of how they relate to the functional differences of the manual haptic modality and the hypothesis of a “haptic bond” between visual and auditory stimuli.

## Introduction

It is well-known that human beings are able to associate stimuli (novel or not) perceived in their environment [1]–[5]. For example, this ability is used by children in reading acquisition when arbitrary associations between visual and auditory stimuli must be learned. Indeed, it is generally agreed upon that reading acquisition consists of two parts: on the one hand, the development of phonological and orthographic representations and, on the other hand, the establishment of associations between these two types of representation [6]–[7]. There is little research devoted to the way in which these associations come about and what there is tends to consider it as an “implicit” process triggered by the learning of letter/sound correspondences. Reading training intervention adheres to this conception [8]–[9]. However, although this type of intervention has a positive effect on reading, its acquisition generally remains slow and difficult. This means several months of formal instruction are necessary before young children grasp the logic of the alphabetic principle and use it [10]–[12].

In light of Bryant and Bradley's work (1985) [13], we assume that one of the difficulties involved in learning how to read relies partly on the establishment of associations between the orthographic representation of a word and the corresponding phonological representation, i.e., between the visual image of the word and its auditory image. In an attempt to overcome this difficulty, a “multisensory” learning method not relying only on the visual and auditory modalities as is traditionally the case, but also on the manual haptic modality, can be used. Indeed, our hands do not simply possess the motor function of moving or transforming the objects in our environment, but also have a highly efficient active perceptual function [14]–[17].

Several studies revealed the positive effects of the visuo-haptic exploration of letters in relief when learning how to read, i.e., learning how to arbitrarily associate visual and auditory stimuli. In these studies, Gentaz and his colleagues [18]–[21] evaluated the effects of two methods intended to help very young children in the understanding of the alphabetic principle. Both interventions proposed several exercises concerning the knowledge of letters (graphemes), the identification of sounds (phonemes) and the letter-sound correspondences. One letter/sound association was studied in each session. The two interventions differed according to the perceptual modalities used to explore the target letters: with the visual modality only in the classic (control) intervention and with both the visual and haptic modalities in the multisensory (experimental) intervention. The letter knowledge, phoneme identification and the decoding of pseudo-words were evaluated before and after the interventions. Among children with a standard level of letter knowledge, results revealed that the performance in the decoding of pseudo-words increased after both interventions but were significantly higher after the multisensory intervention. It should be noted that the knowledge of letters and the identification of phonemes increased similarly after both interventions.

These studies showed that the incorporation of the visuo-haptic exploration of relief letters during a training session, focused on the alphabetic principle, increased its positive effects on decoding skills in kindergarten children. Two complementary hypotheses were proposed to explain these positive effects [18]–[19]. The first hypothesis was based on the addition of the motor information associated inherently with the cutaneous and kinesthetic information generated during the visuo-haptic exploration of visual letters. This multiple coding of the letter may increase the memorization of each letter's shape [22]–[23], (for neural bases [24]–[25]) and would enable a faster activation of the multisensory representation of the letters. As a result, letter identification and then reading ability could be facilitated. The second hypothesis was based on the functional differences of the sensorial modalities involved in interventions [14], [19]. Indeed, vision is characterized by its quasi-simultaneity and is therefore more suitable for processing and representing spatial stimuli such as letters. On the other hand, listening is sequential in nature and is more suitable for processing temporal stimuli such as the sounds of speech. This functional difference could explain why young children have some difficulties in establishing the association between letters, which are processed visually, and sounds, which are processed auditorily. In contrast, the haptic modality shares characteristics with both the auditory and the visual modalities. Even though its functioning is highly sequential in nature, haptic perception is also spatial perception since the exploration in this modality is not linear and subject to a fixed order. The sequential exploration generated by the incorporation of the haptic modality would lead children to process the letters in a more analytical way than when the letters were visually presented. Taken together, the visuo-haptic exploration would help to build a link between the visual processing of the letter and the auditory processing of the corresponding sound; a “haptic bond effect”.

The aim of the present study was to examine whether the addition of the visuo-haptic exploration would lead to more efficient learning of arbitrary associations between visual and auditory novel stimuli in adults as well. This question was not trivial because the characteristics of the visuo-haptic exploration of adults are different from those of young children. First, the study of analytical processes and the integration of different object properties into a unified whole with Garner's (1974) [26] classification research paradigms have provided of some differences in the haptic modality in children and adults, mainly due to the nature of haptic exploration [27]–[29]. Because of partial and poorly organized exploratory procedures, young children proceed to inspect certain properties sequentially, and their classifications are thus based on the dimension they perceived well.

That is why, in free classification tasks, changes due to age seem to operate in the opposite direction to what is usually observed in vision, since the young preferentially make dimensional classifications and not classifications by overall similarity. But adults have reversed results: they classify more by overall similarity than by dimension in haptics, contrary to what is done in vision [30]–[32], because they give priority to the ultimate step of perceptive processing which, in haptics, is the reconstruction of the total object from its elements. Second, a visual dominance is often observed in adults: the bimodal exploration (simultaneous visual and haptic exploration) of spatial properties of shape is not more efficient (when the test is visual) than unimodal visual shape exploration. This visual dominance is not systematically observed in young children [33]–[35].

To examine whether these characteristics of the haptic exploration of adults would influence the positive effect of the addition of this modality, adults were asked to learn 15 new associations between novel visual stimuli and their corresponding sounds in two learning methods which differed according to the perceptual modalities involved in processing the stimuli. Adults used either their visual modality in the “classic” learning method or their visual and haptic modalities in the “multisensory” learning method. The performance in two intramodal (visual and auditory) recognition tests and two intermodal (visuo-auditory and auditory-visual) recognition tests were evaluated immediately after each intervention and one week after. In intramodal tests, adults were asked to find which visual (or auditory) stimulus was previously learned among five alternatives. In intermodal tests, a visual (or auditory) stimulus was presented to the participant who was asked to find its matching sound (or visual) stimulus among 5 alternatives. If the addition of the visuo-haptic exploration is efficient in adults as well and thus helps them to learn more effectively the arbitrary association between visual and auditory novel stimuli, performance in the intermodal recognition tests would reveal traduce this positive effect. The ability to recognize the associations would be above the chance level after both interventions but higher after the multisensory method than after the classic one. If this positive effect is indirectly due to better memorization of visual stimuli (as we hypothesized in previous studies), participants would show a better ability to recognize the visual stimuli after the multisensory method than after the classic one. In all cases, participants would show a similar above-chance ability to recognize the auditory stimuli after both methods. Furthermore, we expect that with a delay between the learning phase and recognition tests, performance will remain stable after the multisensory training method and decrease for the classic one, according to time-dependant consolidation found in some motor memory tasks [36] and the standard forgetting curve in the visual memory.

## Results

### 1 Intramodal recognition tests

Firstly, we examined independently the efficiency of learning of the visual stimuli and auditory stimuli for both groups. Student tests were used to compare the results of each group immediately after the learning phase to “chance level” ( = 1/5 per item = an overall score of 3/15). For the visual stimuli, results showed that performance was significantly different from this chance level in the multisensory (M = 7.3 and SD = 1.9; t(14) = 8.5, p<.05) and classic (M = 8 and SD = 2.9; t(14) = 6.6, p<.05) groups. This means that learning of the visual stimuli had occurred in both groups. Regarding the auditory stimuli, the results showed that the performance in the immediate recognition test was significantly above the chance level in the multisensory (M = 8.87 and SD = 1.13; t(14) = 20.2, p<.05) and classic (M = 8.13 and SD = 2.7; t(14) = 7.38, p<.05) learning methods. This means that learning of the auditory stimuli had occurred for both groups too (Figure S1).

#### 1.1. Visual test

An analysis of variance (ANOVA) was performed on the mean number of visual stimuli correctly recognized, with delay (immediate and delayed recognition) as within-subjects factor and learning methods (multisensory or classic) as between-subjects factor (Figure S1). This analysis did not reveal a main effect of training method [F(1,28) = 0.76 ; p = .39]. The delay increased performance [F(1,28) = 4.46; p<.05; with R^2^ = 0.14], with better performance in delayed recognition (M = 8.7; SD = 2.4) than in immediate recognition (M = 7.6; SD = 2). The interaction between learning method and delay was not significant [F(1,28) = 0.1; p = .75].

#### 1.2. Auditory test

An analysis of variance (ANOVA) was performed on the mean number of auditory stimuli correctly recognized, with delay (immediate and delayed recognition) as within-subjects factor and learning methods (multisensory or classic) as between-subjects factor (Figure S1). This analysis did not reveal a main effect of training method [F(1,28) = 0.03; p = .86], delay [F(1,28) = 1; p = .33] nor learning method×delay interaction [F(1,28) = 1.64; p = .21].

### 2. Intermodal recognition tests

First, we examined the efficiency of learning of associations between visual and auditory stimuli for both groups. Student tests were used to compare the results of each group immediately after the learning phase to “chance level”. In the visual-auditory test, results showed that the immediate performance was significantly above chance level in the multisensory (M = 9.20 and SD = 1.26; t(14) = 18.98, p<.05) and classic (M = 6.67 and SD = 2.9; t(14) = 6.06, p<.05) groups. In the same way, results in the auditory-visual test showed that the immediate performance was significantly above chance level in multisensory (M = 6.4 and SD = 1.80; t(14) = 7.30, p<.05) and classical (M = 5.7 and SD = 2.5; t(14) = 4.14, p<.05) learning methods (Figure S2). This means that learning of the arbitrary associations had occurred in both groups whatever the direction of association (from vision to audition or from audition to vision).

Secondly, an analysis of variance (ANOVA) was performed on the mean number of associations between visual and auditory stimuli correctly recognized, with the direction of association (visuo-auditory and audio-visual) and delay (immediate and delayed recognition) as within-subjects factors and, learning methods (multisensory or classic) as between-subjects factor (Figure S2). This analysis revealed a main effect of training method [F(1,28) = 8.66 ; p<.005, with R^2^ = .24]. Indeed, performance was significantly higher after the multisensory learning (M = 7.12 and SD = 2.17) than after the classic one (M = 5.57 and SD = 2.27). The delay decreased performance [F(1,28) = 14.58; p<.001; with R^2^ = .34], with a higher mean number of correct recognitions immediately after the learning (M = 6.88 and SD = 2.34) than one week later (M = 5.8 and SD = 2.23). There was also a main effect of the direction of association [F(1,28) = 26.38 ; p<.0001; with R^2^ = .49]: Participants performed better in the visuo-auditory recognition test (M = 7.22 and SD = 2.18) than in the audio-visual recognition test (M = 5.47 and SD = 2.19).

The interaction between the method and the direction of association was significant also [F(1.28) = 4.85; p<.05]. The Newmans-Keuls comparisons (with a 0.01 alpha level) revealed that participants after the multisensory learning method recognized more associations in the visuo-auditory recognition test (M = 8.37 and SD = 1.47) than in the audio-visual test (M = 5.87 and SD = 2.03). By contrast, after the classic learning method, the performance observed in the visuo-auditory (M = 6.07 and SD = 2.16) and audio-visual tests (M = 5.07 and SD = 2.26) did not differ significantly. Furthermore, performance in the visuo-auditory test was significantly better after the multisensory learning than after the classic one whereas performance was equivalent for the two groups in the audio-visual test (Figure S3). Interactions between learning method and delay [F(1,28) = 0.1; p = .33] and delay and direction of association [F(1,28) = 0.05; p = .83] were not significant. Neither was interaction between learning method, delay and stimulus type [F(1,28) = 2.42; p = .13].

## Discussion

This study examined whether the addition of the haptic modality would lead adults to more efficient learning of arbitrary association between visual and auditory novel stimuli. In addition, we hypothesized that this enhancement could be due to a better memorization of shapes. To test these hypotheses, adults were asked to learn 15 associations between novel visual stimuli and their corresponding sounds with two learning methods which differed according to the perceptual modalities involved to process the visual stimuli. The participants used their visual modality in the “classic” method and their visual and haptic modalities in the “multisensory” method.

The first result was that the performance in the visuo-auditory recognition test was above chance after both methods but was better after the multisensory learning method than after the classic one. The addition of haptic exploration of visual novel stimuli seems to help adults to associate more shapes and sounds than does visual exploration only. This result was consistent with the results observed in ordinary children, as reported in the introduction. But, contrary to our hypotheses, the performance in the audio-visual recognition test (i.e., the reverse direction) was similar after using both learning methods. Furthermore, an asymmetry appeared in the intermodal recognition tests for the multisensory group. Indeed, participants recognized more associations in the visuo-auditory recognition test than in the audio-visual one. It should be noted that asymmetries were often observed in crossmodal tasks like between vision and touch in infants, children and adults ([37]–[39]) and their explanations are still in debate (for a review [15]).

According to Ernst and Bulthoff [40], one determinant of where crossmodal convergence of object information occurs is which modality provides the most accurate information about objects. Usually, humans obtain most of their information about objects from vision for which shape is the most salient attribute. In our research, because we used arbitrarily related multimodal information (shapes and sounds), both visual and auditory modalities involved in learning delivered different relevant information (spatial and temporal). Thus, the crossmodal memory could be maximized (“sensory combination”) and participants were able to recognize clearly shapes from sounds and vice versa. On the other hand, when we added the haptic modality in the training phase, it is possible that redundant signals about spatial information on the visual stimuli, provided by both visual and haptic modalities, increased the percept reliability (“sensory integration”). First, it could explain why the multisensory group obtained better performance than the classic one. Secondly, because of competition from more salient modality-specific information (spatial vs. temporal), it could generate an asymmetric crossmodal performance.

The second set of important results showed an equivalent mean number of visual stimuli recognized by participants after both training methods. The haptic effect observed in the intermodal task cannot be simply explained by a better memorization of the visual stimuli. However, Pascual-Leone and Hamilton assume that relevant inputs from senses are exploited to execute particular processing tasks successfully [41]. It could be speculated, because of the nature of the task (to recognize learned shape among unlearned ones), that the visual modality alone is recruited and provides enough reliable spatial information to perform the task as accurately in both learning groups. We could also hypothesize that the shape percept is improved by additional information collected via the haptic exploration, which may improve recognition speed. Then, the intramodal recognition test used in the present experiment constituted too global a measure of the shape knowledge because it did not take into account the speed of shape recognition.

Finally, we noticed a slight improvement of performance in the visual intramodal recognition test after a week of delay. This result was not expected because we believed that performance remained stable in the multisensory training and decreased in the classic one because of the potential effect of the haptic exploration on the memorization of shapes. This result is probably a false positive because, on the delayed recognition test, the participants of both groups benefited further from having seen the trained shape a second time during the immediate intermodal recognition test.

In conclusion, the present study underlined the positive effects of the addition of visuo-haptic exploration in learning of arbitrary associations between novel visual and auditory stimuli in adults (for review about haptics in education [42]). Although the mechanisms of its action are still in debate, haptic exploration seems to play a role in “bonding” between visual and auditory stimuli in young children as well as in adults, whether or not the characteristics of visuo-haptic exploration of adults are different from those of young children.

## Methods

### 1. Participants

Thirty monolingual French adult students took part in this experiment (Table 1). The participants in the two learning methods were matched on each of the following criteria: age and Raven test (t test: p>.25). There were 15 adults in each group. The present study was conducted in accordance with the Declaration of Helsinki. It was conducted with the understanding and the written consent of each participant which was obtained and was approved by the local ethic committee of the LPNC (CNRS and University of Grenoble 2).

**Table 1:** Characteristics of participants in each training group.

Category	Classic group	Multisensory group
**Age: M**	20 years and 6 months (from 18 to 28 years)	20 years and 6 months (from 18 to 24 years)
**Raven score: M (SD)**	10.8 (5.7)	10.4 (3.9)

### 2. Stimuli

#### 2.1. Visual/haptic stimuli

The visual and haptic stimuli were signs derived from the Japanese katakana alphabet. All thirty five stimuli were created using graphical software. Fifteen of these were selected at random as visual and haptic stimuli (Figure S4) to learn and others (Figure S5) were used as visual distractors. The visual stimuli were printed on paper cards for the classic group and the haptic stimuli were cut into foam and then glued on paper cards for the multisensory group. The haptic stimuli were not used in the classic learning group to avoid spontaneously manual explorations which were forbidden in this condition. The visual and haptic stimuli used in learning sessions were the same for the two groups (average dimensions were about 7.5×11.5×0.5 cm).

#### 2.2. Auditory stimulus

Thirty five sound stimuli were created with software that generates a computerized voice (Microsoft Sam). These sound stimuli were constituted of sequences of two or three letters/sounds co-articulated so, undistinguishable individually. The combinations of these computerized sounds were chosen to be without meaning. The duration of each sound stimulus was on average of 500 ms. Theses sound stimuli were then converted to the Mp3 format and played using the Winamp player. Fifteen were selected at random as stimuli to be learned and others used as distractors in the auditory recognition test (each visual distractor was presented twice). These sound stimuli were presented to the participants through headphones (Sony MDR-V150).

#### 2.3. Association between visual/haptic and auditory stimuli

The association between the 15 visual and auditory stimuli was randomly determined. Once the associations were created, they remained the same across participants. The presentation order of these associations was randomized across participants of both groups.

### 3. Procedure and experimental conditions

Two groups, each composed of 15 participants, were constituted. Each group was assigned to a specific method: a classic (visual-auditory) learning method or a multisensory (visual-auditory-haptic) learning method. Each learning method was presented to each participant in a single session. After this learning phase, each participant performed the same four recognition tests immediately after intervention and one week later.

#### 3.1. The two learning methods

In the visual-haptic-auditory group (multisensory method), each participant had to learn the 15 associations (the visual stimuli and their corresponding sounds) by using both the visual, haptic and auditory modalities. For each association, the participants explored the visual stimulus using their eyes and hands and simultaneously heard the corresponding sound. It should be noted that the visuo-haptic exploration of haptic stimulus was obligatory and unguided. Because the duration of visual and haptic exploration was about 10 seconds, and in order to equal the time of presentation of both the visual-haptic and auditory stimuli, the sound was repeated three times with a 3s inter-stimuli interval. This procedure was repeated for each of the 15 associations. In the visual-auditory group (classic method), the experimental procedure was the same as for the visual-haptic-auditory group, except that the participants used only their visual and auditory modalities to learn the associations.

#### 3.2. The four recognition tests

After this learning phase, there were four recognition tests. Each participant performed two intramodal tests, in random order, followed by two intermodal tests, also in random order. Intramodal tests were stimulus recognition tests (visual and auditory) and intermodal tests, stimuli association recognition tests (visual-auditory and auditory-visual). Tests were given immediately after both methods (immediate recognition) and the other a week later (delayed recognition). No feedback was given.

In the visual intramodal recognition test, the participants had to find which visual stimulus was previously learned among five alternatives (1 target and 4 distractors). For each of the 15 learned visual stimuli, the participants were given an A4-sized sheet with five printed shapes. The participants were asked to “circle on the paper with a pen” the shape they recognized as being the target stimuli. The presentation order of the visual stimuli and the spatial position of the “learned” stimuli among distractors were randomized. In sum, there were 15 visual responses for each participant. In the auditory intramodal recognition test, the participants had to recall which one of five sounds (1 target and 4 distractors) was previously learned. The presentation order of the stimuli and the temporal position of the “learned” stimuli among the distractors were randomized. In sum, there were 15 auditory responses for each participant.

In the visuo-auditory intermodal recognition test, a visual stimulus was presented to the participant who was asked to find its matching sound among 5 alternatives (1 target and 4 other sounds). These four auditory stimuli were presented in the previous learning phase but were linked to different visual stimuli. In sum, there were 15 visuo-auditory responses for each participant. In the audio–visual intermodal recognition test, a sound was presented to the participant who then had to find its corresponding visual stimulus among 5 alternatives (1 target and 4 other visual stimuli). These four visual stimuli were presented in the learning phase but were linked to different auditory stimuli. In sum, there were 15 audio-visual responses for each participant. In total, there were 60 responses per participant.

It should be noted that the two intramodal recognition tests and the two intermodal recognition tests were different in nature: in the intramodal tests, the participants were asked to recognize one target (learned stimulus) among novel distractors (unlearned stimuli) whereas in the intermodal tests, they were asked to recognized one target (learned stimulus) among others learned (i.e., familiar) but not relevant stimuli.

## Supporting Information

Figure S1 Mean number and standard error of visual and auditory stimuli correctly recognized (maximum 15) as function of learning method and delay. The dotted line corresponds to the level of chance performance.
(2.11 MB TIF)

Figure S2 Mean number and standard error of visuo-auditory and audio-visual associations correctly recognized (maximum 15) as function of learning method and delay. The dotted line corresponds to the level of chance performance.
(2.10 MB TIF)

Figure S3 Mean number and standard error of visuo-auditory and audio-visual associations correctly recognized (maximum 15) as function of learning method.
(2.11 MB TIF)

Figure S4 The 15 visual/haptic stimuli used in both learning methods.
(0.07 MB TIF)

Figure S5 Eight examples of stimuli used as visual distractors in immediat and delayed intramodal recognition tests.
(0.06 MB TIF)
