# Supplementary figures and images for: Learning of Arbitrary Association between Visual and Auditory Novel Stimuli in Adults: The “Bond Effect” of Haptic Exploration
Source: PLoS One. 2009 Mar 16;4(3):e4844. doi: 10.1371/journal.pone.0004844 (PMC2653648; doi:10.1371/journal.pone.0004844)

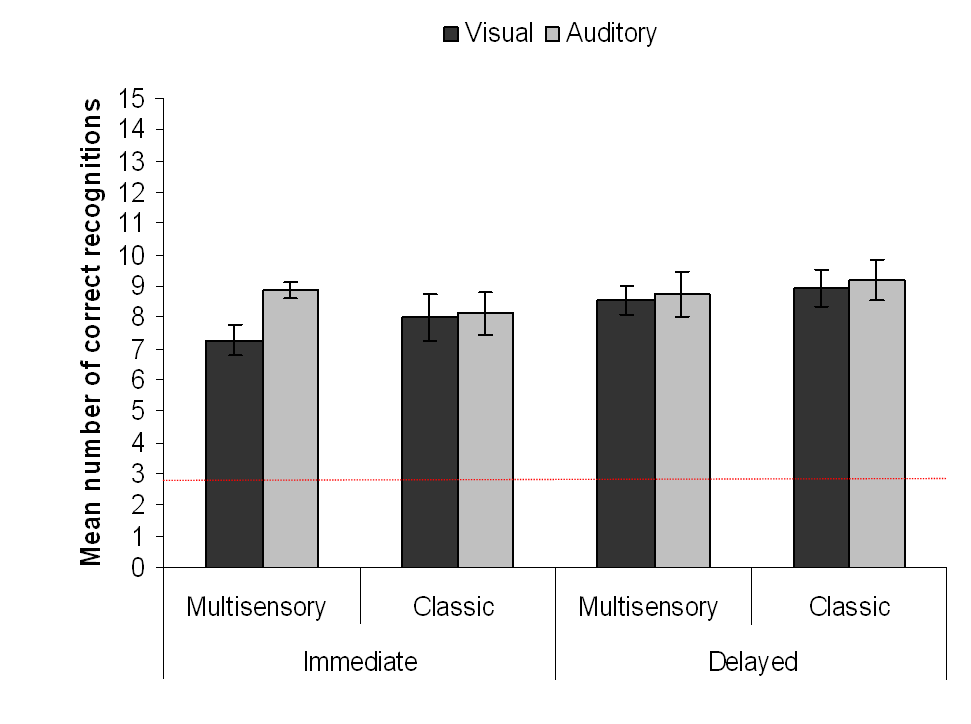

Supplement: Figure S1 — Mean number and standard error of visual and auditory stimuli correctly recognized (maximum 15) as function of learning method and delay. The dotted line corresponds to the level of chance performance. (2.11 MB TIF) [file pone.0004844.s001.tif]

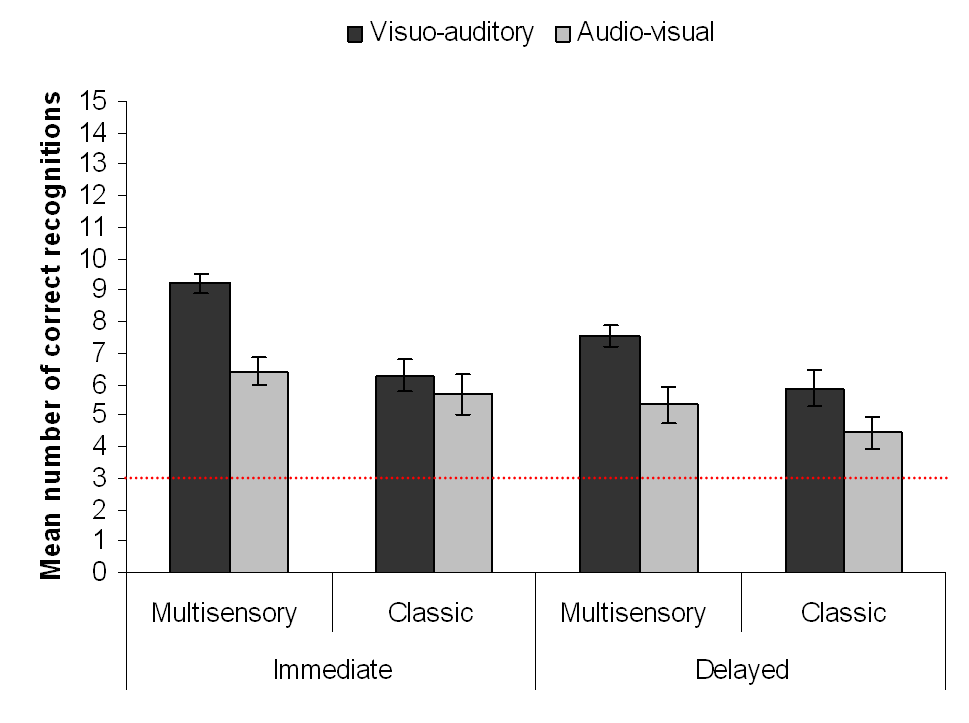

Supplement: Figure S2 — Mean number and standard error of visuo-auditory and audio-visual associations correctly recognized (maximum 15) as function of learning method and delay. The dotted line corresponds to the level of chance performance. (2.10 MB TIF) [file pone.0004844.s002.tif]

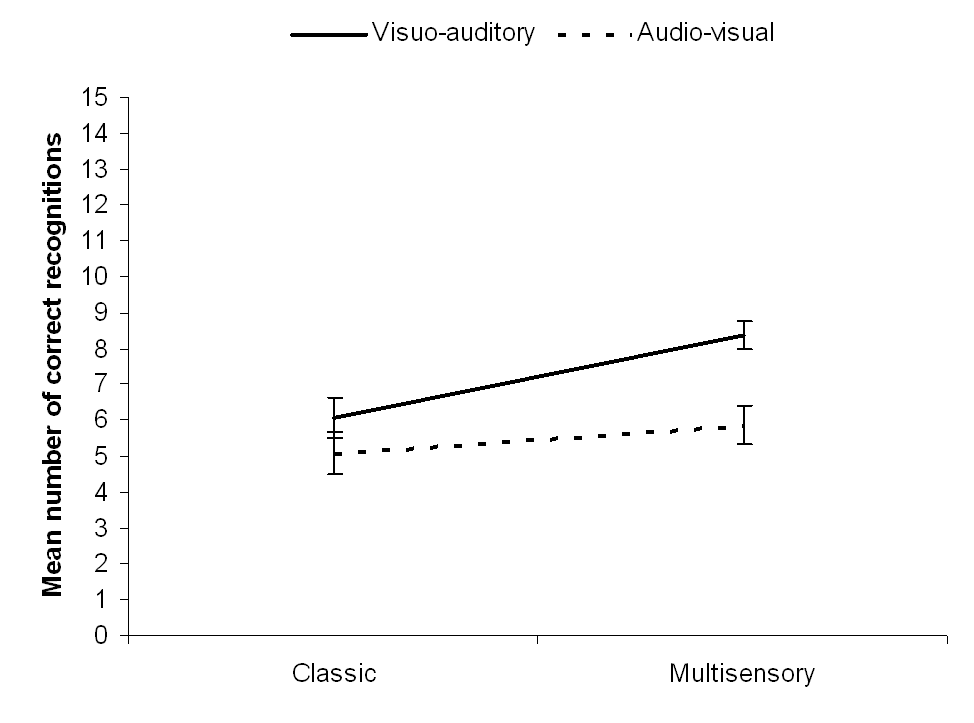

Supplement: Figure S3 — Mean number and standard error of visuo-auditory and audio-visual associations correctly recognized (maximum 15) as function of learning method. (2.11 MB TIF) [file pone.0004844.s003.tif]

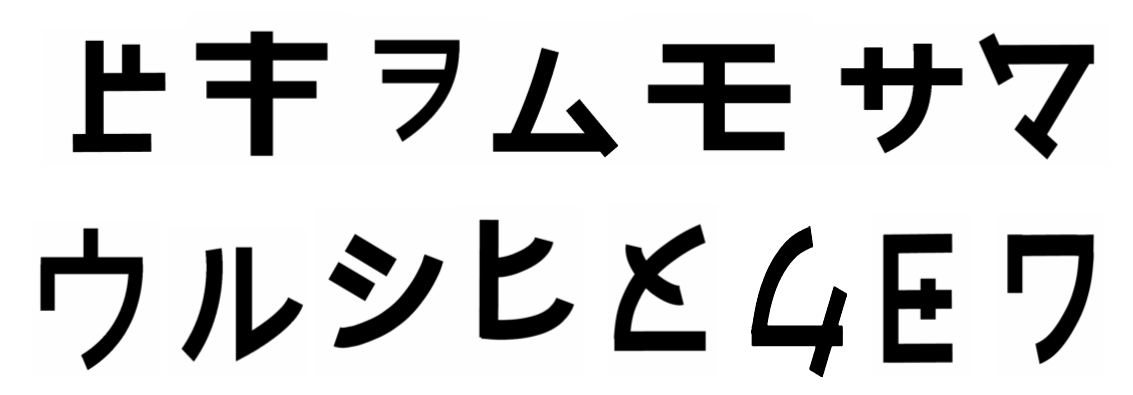

Supplement: Figure S4 — The 15 visual/haptic stimuli used in both learning methods. (0.07 MB TIF) [file pone.0004844.s004.tif]

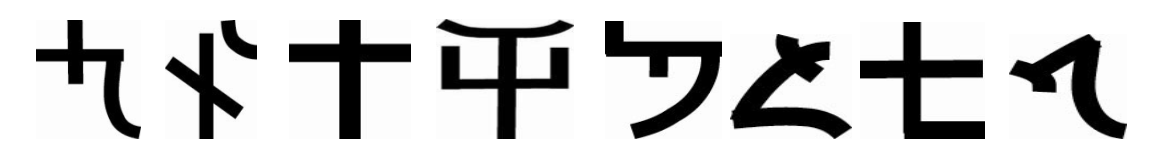

Supplement: Figure S5 — Eight examples of stimuli used as visual distractors in immediat and delayed intramodal recognition tests. (0.06 MB TIF) [file pone.0004844.s005.tif]
